# Supplementary material for: Cancer patient survival can be parametrized to improve trial precision and reveal time-dependent therapeutic effects
Source: Nat Commun. 2022 Feb 15;13:873. doi: 10.1038/s41467-022-28410-9 (PMC8847344; doi:10.1038/s41467-022-28410-9)
Supplement: Supplementary file 5 — Supplementary Dataset 2 [file 41467_2022_28410_MOESM5_ESM.zip › Supplementary Data File 2/Alpha vs beta plots- Fig 5, S8/Surrogate Event-Free Survival Cancer Type Met Status.pdf]

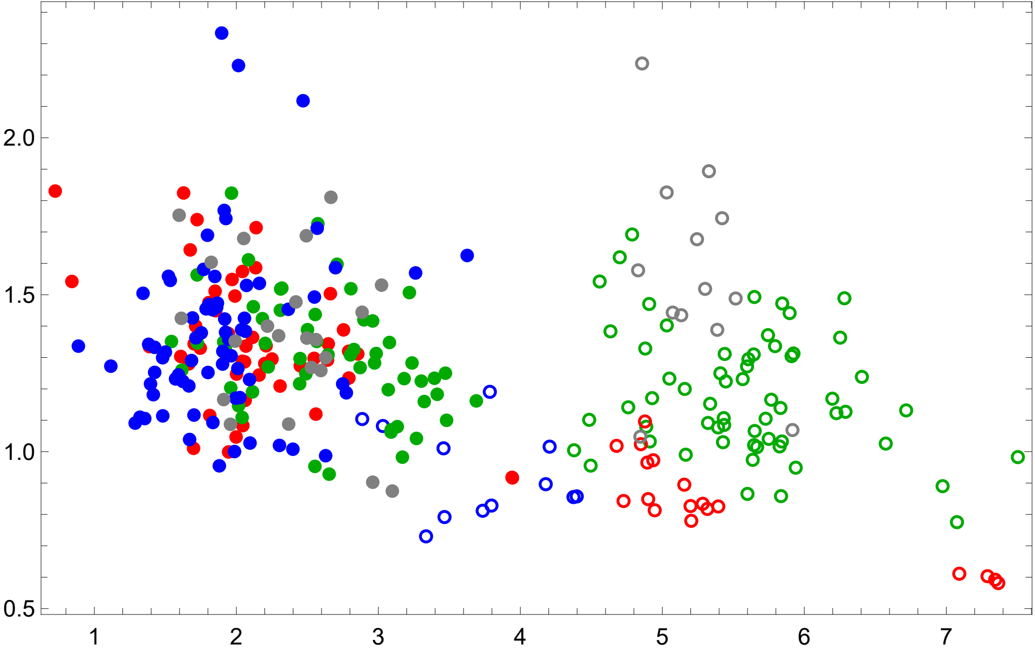
2.0
1.5
1.0
0.5
1
2
3
4
5
6
7
